# Assessment of Heavy Metals and Related Impacts on Antioxidants and Physiological Parameters in Oil Refinery Workers in Iraq

**DOI:** 10.5696/2156-9614-11.31.210907

**Published:** 2021-08-17

**Authors:** Mohammed A. Ajeel, Akram A. Ajeel, Aws Maseer Nejres, Riyam Ameen Salih

**Affiliations:** 1 College of Pharmacy, University of Mosul. Iraq; 2 Ministry of Education/Salah El-Din Education Directorate, Iraq; 3 Al-Dour Technical Institute, Northern Technical University, Iraq

**Keywords:** heavy metals, antioxidants, physiological parameters, refinery workers, atomic absorption spectrometry

## Abstract

**Background.:**

Some heavy metals can be harmful to human health in elevated doses such as zinc (Zn) and magnesium (Mg), while others such as lead (Pb), cadmium (Cd), mercury (Hg), and copper (Cu) have harmful consequences to health even in small doses. Heavy metals and additives are incorporated into crude oil to enhance performance.^3^,^4^,^5^ Crude oil is well known to contain heavy metals like Cu, Cd, Ni and Pb.^3^,^6^,^7^

**Objective.:**

The current study aimed to assess levels of heavy metals and the impact of these metals on antioxidant levels and physiological variables in the serum of oil refinery workers in Iraq.

**Methods.:**

Heavy metals such as Pb, Cd, Hg, Zn, Cu and Mg were assessed in the serum of a sample of refinery workers (N=40) and a control group (N=20) using atomic absorption spectrometry (AAS). Additionally, levels of malondialdehyde (MDA), δ-aminolaevulinic acid dehydratase (ALAD) and total antioxidant capacity (TAC), and physiological variables such as blood urea, serum creatinine, glutamate-oxaloacetic transaminase (GOT), glutamate-pyruvic transaminase (GPT), and gamma-glutamyl transferase (GGT) were measured to assess impact of these heavy metals.

**Results.:**

Mercury, Cd, and Pb were significantly elevated in the refinery worker group in comparison with the control group, while the levels of Zn, Cu, and Mg were significantly lower in the refinery worker group compared to the control group. There was a significant difference between the control group and the worker group for most of the antioxidants and functional variables. Total antioxidant capacity (TAC), δ-aminolevulinic acid dehydratase (ALAD), and malondialdehyde (MDA) were significantly lower in the worker group while blood urea, serum creatinine, glutamate pyruvate transaminase (GPT), and gamma-glutamyl transferase (GGT) showed a significant elevation in the workers' group. Glutamic oxaloacetic transaminase (GOT) showed no significant difference between the control group and the worker group.

**Conclusions.:**

Refinery workers are at increased risk of having higher serum levels of Pb, Cd, and Hg compared to controls which can lead to an increase in oxidative stress, decrease in TAC, and decrease in the essential trace elements Zn, Cu and Mg.

**Participant Consent.:**

Obtained

**Ethics Approval.:**

This study was approved by the ethics committee within the Nineveh Health Department, Mosul, Iraq.

**Competing Interests.:**

The authors declare no competing financial interests.

## Introduction

Heavy metals can be harmful in elevated doses to human health such as zinc (Zn) and magnesium (Mg), while others such as lead (Pb), cadmium (Cd), mercury (Hg), and, copper (Cu) have harmful consequences to health even in small doses.^1^,^2^ Several studies have shown that accumulated heavy metals in the body can damage nerves, kidney, liver, blood composition and other organ systems.^1^,^2^ Heavy metals and additives are incorporated into crude oil to enhance performance.^3^,^4^,^5^ Crude oil is well known to contain heavy metals like Cu, Cd, Ni and Pb.^6^,^7^ Akpoveta *et al.* found high levels of Pb and Cu in refined petroleum, and concluded that this posed serious ecological and environmental threats in the vicinity where these products are used.^8^ In addition, refined petroleum products are ubiquitous and can negatively affect the biological systems of the human body.

Oxidative stress is an imbalance between free radical production capacity and antioxidants.^9^ Cell damage resulting from free radicals plays a key role in the progression of disease and aging processes.^10^ The negative effects of oxidative stress affect several structures of the cells, such as lipids, proteins, lipoproteins, membranes, and deoxyribonucleic acid (DNA).^11^,^12^,^13^,^14^ Oxidative stress leads to the formation of conjugated diene compound and malondialdehyde (MDA), which are known for their mutagenic and cytotoxic activity. Proteins may undergo conformational changes under the oxidative stress effect that may cause impairment and/or loss of their activity.^13^,^15^

Lipid peroxidation spreads by a radical chain reaction, affecting a large number of lipidic molecules in the cell.^15^ The DNA and epigenetic information are prone to the lesions of oxidative stress.^16^,^17^ Oxidative damage to DNA could contribute to tumor onset when a certain amount of modifications on the DNA structure happens, such as strand breaks, DNA-protein cross-links, sugar and base lesions, and base-free sites.^18^,^19^,^20^ Many factors can lead to increased exposure to free radicals, including exposure to heavy metals.^21^ The current study aimed to assess levels of some heavy metals in blood, and to evaluate the impact of these metals on antioxidant levels and physiological variables in the serum of the oil refinery workers.

## Methods

**Figure 1:**
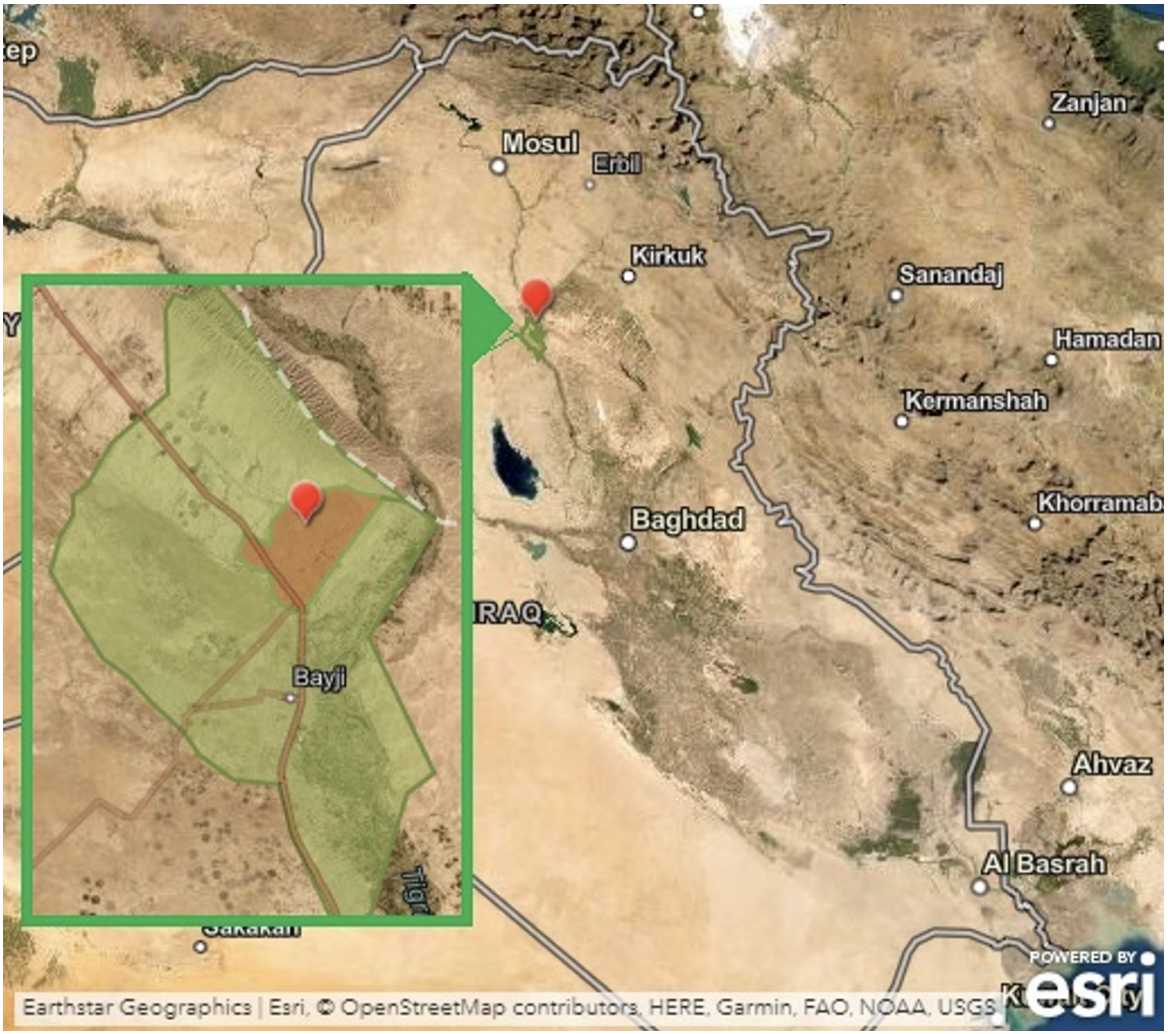
Map of study area

The study was conducted in Bayji, a major industrial city in northern Iraq *(Figure 1).* Sampling was performed at the Bayji oil refinery. The refinery is far from industrial activities or agricultural activities involving the use of heavy metal-containing fertilizers, suggesting that heavy metal distributions are solely attributed to refinery activities. The city of Bayji is located about 34 miles from Tikrit. Tikrit is the center of Salah-Addin province and is characterized by higher levels of anthropogenic activities that may increase accumulation of heavy metals. Baiji is nearly the same size as Tikrit, yet most of its area is rural.

**Abbreviations**
*ALAD*: δ-aminolevulinic acid dehydratase
*MDA*: Malondialdehyde
*TAC*: Total antioxidant capacity

Non-smoking refinery workers (n = 40 males) were selected through convenience sampling. After consent were taken, the questionnaire was delivered to each volunteer and blood samples were taken.

All of the workers working in direct contact with the refining process were males, so all of the study participants were male as a result. Twenty subjects were recruited from urban areas away from any industrial activity or highways as a control group. The control group resembled the refinery workers group in age and gender.

A questionnaire *(Supplemental Material)* included data on health problems, age, daily working hours, industry working experience, and personal protective equipment.^22^ The questionnaire data was collected by the staff of the Baiji refinery health center, trained by the researchers in questionnaire administration. Ethics approval for the study was obtained from the Ethics Committee of the Nineveh Health Department, Mosul, Iraq. The study objective was explained to all participants and written consent was obtained from all study subjects.

### Blood sample collection

Ten (10) ml of peripheral blood was collected from all study subjects. Samples were divided into two blue EDTA tubes (ethylenediaminetetraacetic acid). One EDTA tube (4 ml) was used for assessing heavy metals while the other EDTA tube was centrifuged and blood serum was isolated. The EDTA tubes were inverted 8–10 times to prevent clotting, then each tube was labeled and kept at 4°C.^23^

### Blood serum digestion

For digestion of blood samples, 15 ml of (10%) Triton X-100, 5 ml of ammonium dihydrogen phosphate ((NH_4_)(H_2_PO_4_)) (20%), and 1 ml of concentrated nitric acid (HNO3) was mixed in a 500-ml volumetric flask.^24^ The volume was completed to 500 ml with deionized water. Next, 9 ml of the solution was mixed with 4 ml of each blood sample. After 15–20 minutes of incubation, the mixture was heated and evaporated to near dryness. The incubation, heating and evaporation process was then repeated. After cooling, 2 ml of sodium hydroxide NaOH was added for pH neutralization.

### Determination of metal concentrations

Standard solutions for magnesium (Mg), copper (Cu), zinc (Zn), mercury (Hg), cadmium (Cd), and lead (Pb) were used for atomic absorption spectrophotometry (AAS) (AA-6300, Shimadzu, Japan.) Quality assurance/quality control for AAS was performed according to the manufacturer's instructions manual. Standard solutions were prepared from a stock solution of 1 g /L for each of the metals through several successive dilutions by distilled water. The results were used for instrument calibration. Then, heavy metals in each digested blood sample were measured and recorded for the current study analysis.

### Physiological variables

Blood urea and serum creatinine were used to evaluate the functional state of the kidney. Glutamate-pyruvic transaminase (GPT), glutamate-oxaloacetic transaminase (GOT), and *gamma*-*glutamyl transferase* (GGT) were used as indicators of liver function. All tests were performed using a semi-auto biochemical analyzer (SK3002b).

### Antioxidant variables

A double beam spectrophotometer (Fisher Scientific, Jenway™ 6850/230V) was used for analysis of antioxidant variables. For quality assurance/quality control of the spectrophotometry, a standard stock solution was made using potassium permanganate (KMnO4) 0.072g in a 250 ml standard flask. A series of concentrations (1, 2, 4, 6, 8, 10, 20, 40 mg/dm^3^) working solutions were prepared of the stock solution of potassium permanganate using an appropriate amount of deionized water.

The absorbance of each solution was measured at 480, 526, and 580 nm using the spectrophotometer. To determine the maximum absorption wavelength at the different concentrations, the spectrum of KMnO4 was performed by plotting the data of absorbance versus wavelength. The calibration curve was made by fitting the absorbance versus concentration at the maximum absorption wavelength. Ringbom–Ayre's plot was performed by fitting transmittance versus concentration and transmittance versus the logarithm of concentration. An R^2^ value close to 1 considered to be an indication of the instrument functionality and the accuracy of the results.^25^

#### Total antioxidant capacity (TAC) measurement

The ferric-reducing ability of plasma (FRAP) procedure, proposed by Koracevic *et al.*,^23^ was utilized to measure total antioxidant capacity (TAC). A standardized iron (Fe)–EDTA solution reacts with hydrogen peroxide by a Fenton-type reaction, leading to the hydroxyl radicals formation. Then thiobarbituric acid (TBA), a reactive acid, was released as a result of reactive oxygen from the Fenton reaction degrade benzoate. Antioxidants from the serum sample cause TBA production to be suppressed which leads to decreasing color intensity. Absorption at wavelength of 532 nm will then be reduced as a result of decreased color intensity.

#### Malondialdehyde measurement

Malondialdehyde (MDA) level was measured using Buege and Aust's method.^26^ In this method, biological samples (e.g., plasma, erythrocytes or other tissues) are heated with TBA in a trichloroaceti–hydrochloric acid (TCA–HCl) mixture for 15 min. Colored products were measured at 535 nm using a double beam spectrophotometer.

#### Blood δ-aminolevulinic acid dehydratase measurement

Blood δ-aminolevulinic acid dehydratase (ALAD) activity was assessed according to Berlin and Schaller's procedure^27^ which incubates ALAD with an excess amount of d-aminolevulinic acid. The formed porphobilinogen was mixed with modified Ehrlich's reagent then measured at 532 nm using a double beam spectrophotometer.

#### Statistical analysis

Statistical analysis was performed using Statistical Package for the Social Sciences (SPSS) version 23. The results were expressed in the tables as mean ± standard deviation (SD). Mean values of refinery workers and the control group were compared using Student's t-test.

## Results

Table 1 presents characteristics of the study participants (refinery workers and control group), health status and working profiles. Personal protective equipment included safety glasses and respirators and face masks. Self-reported health problems related to work activities in the study population included eye and respiratory symptoms, especially allergic symptoms of the eye, occupational asthma and allergic rhinitis.

**Table 1:** Participant Characteristics

**Characteristics**		**Refinery workeny(n=40)**	**Control (n=20)**
Age (years)		30 ± 10	30 ± 10
Self-reported health problems % (n)	Occupational asthma	21	None
	Allergic rhinitis	14	
	Eye allergy	23	
Years working in the industry		12 ± 5	0
Daily working hours		6 ± 1	0
Protective equipment (%)	Respirators or face masks	28	None
	Safety glasses	40	None

Table 2 shows the mean concentration of heavy metals in the blood of the study participants. Half of the measured heavy metals (Hg, Cd and Pb) in the refinery worker group showed a significant increase compared with the control group, while the other half (Zn, Cu, and Mg) were significantly higher in the control group.

**Table 2:** Heavy Metal Levels in Workers and Controls

**Variable**	**Pb (μg/ml)**	**Cd (μg/ml)**	**Hg (ng/ml)**	**Zn (μg/ml)**	**Cu (μg/ml)**	**Mg (μg/ml)**
Control n=20	0.208 ± 0.032	0.026 ± 0.008	16.8 ± 1.837	0.925 ± 0.076	0.819 ± 0.042	*16.2 ± 1.243*
Worker n=40	0.628 ± 0.033	0.046 ± 0.011	34.54 ± 3.854	0.562 ± 0.015	0.316 ± 0.073	*14.94* ± *0.932*
P value	<0.01	<0.01	<0.01	<0.01	<0.01	*< 0.05*

Results are expressed as mean ± standard deviation.

Table 3 shows the mean concentrations of antioxidants and functional variables of studied refinery workers and controls. There was a significant difference between the control group and the refinery worker group for most of the antioxidants and functional variables. Total antioxidant capacity, ALAD, and MDA were significantly lower in the refinery worker group, while blood urea, serum creatinine, GPT, and GGT showed a significant elevation in the refinery worker group. Glutamic oxaloacetic transaminase showed no significant difference between the control group and the refinery worker group.

**Table 3:** Anti-oxidant and Functional Variables of Workers and Controls

**Variable**	**Control**	**Workers**	**P. value**
TAC (mmol/L)	2.1 ± 0.3	1.3 ± 0.2	<0.05
ALAD (μmol)	18.2 ± 0.9	24.9 ± 1.2	<0.05
MDA (μmol/L)	5.7 ± 0.4	2.2 ± 0.3	<0.05
Blood urea	25.7 ± 3	35.6 ± 6	<0.05
Serum creatinine	0.87 ± 0.17	1.1 ± 0.21	<0.05
GOT	28 ± 2	31 ± 3	>0.05
GPT	30.7 ± 2.8	44.2 ± 2.3	<0.01
GGT	23.5 ± 1.3	34 ± 4	<0.01

Results are expressed as mean ± standard deviation.

Abbreviations: TAC, total antioxidant capacity; ALAD, δ-aminolevulinic acid dehydratase; MDA, malondialdehyde; GOT, glutamic oxaloacetic transaminase; GPT, glutamate pyruvate transaminase; GGT, gamma-glutamyl transferase.

## Discussion

The results of the current study demonstrated a correlation between increased serum concentrations of Pb, Hg, Cd and altered TAC in refinery workers. These heavy metals have a unique affinity for electron–sharing that could form covalent attachments.^28^ These covalent attachments are mainly formed between sulfhydryl groups of proteins and heavy metals.^29^ Metal interaction with glutathione (GSH) metabolism is a critical step in the metal's toxic responses.^30^

Previous studies have indicated that heavy metal toxicity in workers could be clearly seen as an ROS increase that increases MDA levels and lowers TAC levels. The increase in ROS production leads to increases in lipid peroxidation and the production of MDA; which is a good oxidative stress indicator.^31^,^32^,^33^

Zinc is an essential component of antioxidant enzymes. The antioxidant properties of zinc can be presented by two mechanisms. One involves reducing the protein's essential sulfhydryl group susceptibility to oxidation and the other is competing for binding sites with Cu, Fe and other pro-oxidant metals.^34^ As shown in Table 2, there is a low serum Zn level which might be the cause of reduced levels of antioxidant enzymes.^35^

The low concentration of Cu in the serum of refinery workers may be due to the hydroquinone interaction, a benzene metabolite, with Zn and Cu, which are cofactors for the SOD enzyme. Therefore, it displaces Cu from the enzyme. The ROS generated as a result of Cu reaction with other compounds results in the lipid oxidation chain reaction initiation. Copper deficiency thus increases the peroxidation processes by two-fold.^36^

Oxidative stress is induced through free radical increases and/or disturbance of the redox system among refinery workers. It is possible that a low Mg level simply reflects excessive sweating, low Mg intake, or is due to Mg interaction with other heavy metals. A decreased magnesium level can be also justified by the high utilization rate of this metal, as this metal is utilized for protection toward oxidative stress.^37^

The ALAD is an enzyme that contains thiol groups that are susceptible to oxidation. Inhibition of ALAD activity is one of the most sensitive indicators of lead exposure. In this study, ALAD levels in the whole blood of refinery workers were significantly decreased. Therefore, the results of whole blood ALAD activity were decreased as a result of the sulfhydryl group oxidation of the ALAD enzyme by ROS and other oxidants which are generated in refinery workers. The decline in ALAD level among the refinery workers could be due to sulfhydryl group oxidation of this enzyme by Hg; or Hg might be competing with the Zn within the ALAD leading to its inactivation.^38^,^39^

The results clearly showed that levels of trace elements Cu, Zn, and Mg were significantly lower in refinery workers compared with controls. Low levels of Cu, Zn, and Mg in the refinery workers could be a result of an adaptive response to oxidative stress increment and free radical generation as a result of exposure to gasoline and heavy metals in gasoline.^40^

Mercury is known to change some trace element function and metabolism, such as Cu, Mg, Se, and Zn through competing for the binding sites of these trace elements within the human body. Therefore, interference with the absorption of some essential elements in the digestive system could be the reason for the deficiency of these essential elements.^35^

The levels of heavy metals in the present study were lower than those found in many other studies in Iraq. Several studies have reported that heavy metals levels are very elevated in the Iraqi population and the effect of this elevation can appear as serotonin imbalance, thyroid gland function imbalance and even cancer in some cases.^41^,^42^,^43^,^44^

The present study showed a significant increase in the functional variables of the liver and the kidney (Table 3). Most heavy metals exhibit harmful effects at high concentrations on several biological systems such as energy metabolism-related organs, ion transporters, the respiratory system, reproductive system, central nervous system (CNS), cardiovascular system, and other vital organs like the kidney, liver, and lungs.^45^,^46^

Oxidative stress results from heavy metal exposure (such as Hg and Pb) and a decrease in the serum level of essential metals (such as Zn and Se). As a result, the redox status of the cell can be shifted, damaging biomolecules like nucleic acid, lipids, and proteins as well as some organs like the kidney, the liver, and the CNS.^47^,^48^

The liver contains a divalent transporter protein system that transports some trace elements like Fe, Zn, Pb, and Cu. The level of this transporter protein decreases in the liver when some of the transported element levels are low, giving some sort of protection to the liver, but will be increased in the cells of the kidney as an excretion mechanism. This mechanism could harm the kidney, and consequently increase the level of kidney functional variables.^49^

When heavy metals enter the body, they compete with essential trace elements and spend their nephrotoxic consequence through oxidative stress induction, apoptosis mediated by Ca^+2^, and ROS, as well as mitochondrial dysfunction. Heavy metals (except arsenic) alter the permeability and absorption of epithelial cells in the kidney, causing kidney dysfunction and proteinuria. The antioxidant characteristics of Zn could be potent in alleviating the acute effect of heavy metal exposure.^50^

The present study has some limitations. All of the workers involved in the refining process that are in contact with petroleum refining processes were male because of the social and geographical habits in the Middle East generally, and in Iraq specifically. However, this should not affect the generalizability of the results, as heavy metals (at a certain levels) negatively impact all biological systems.

## Conclusions

The present study found evidence for a strong correlation between increased serum concentrations of Pb, Hg, Cd and altered TAC and decreased serum level of the essential trace elements Zn, Cu, and Mg, adversely affecting the renal and liver functional states of refinery workers.
