# Supplementary material for: Assessment of Heavy Metals and Related Impacts on Antioxidants and Physiological Parameters in Oil Refinery Workers in Iraq
Source: J Health Pollut. 2021 Aug 17;11(31):210907. doi: 10.5696/2156-9614-11.31.210907 (PMC8383791; doi:10.5696/2156-9614-11.31.210907)
Supplement: Supplementary file 1 [file Nejres_Supplemental_Material.docx]

**Supplemental Material**

**HEAVY METALS STUDY QUESTIONNAIRE**

Questionnaire for persons who entered as samples in the research “**Assessment of certain heavy metals level and their impact on antioxidants and physiological parameters in oil refinery workers”** after obtaining their personal consent.

# Gender ---------------------------------, Age --.

1. Do you work in contact with the petroleum refining process? [ ] Y [ ] N
2. If the answer to the previous question is yes, how much time do you spend at work each day?

# ------------------------------------------------------------------------

1. How long have you been employed in this job?

# -------------------------------------------------------------------------

**Check ‘Yes’ or ‘No’ in response to the following questions:**

[ ] Y [ ] N 1) Are you a smoker ? Did you previously quit smoking? (smoking quit period) .

[ ] Y [ ] N 2) Have you used personal protective equipment? If so, what kind?

.

[ ] Y [ ] N 3) Have you had any recurring health problems during your employment? If so, please describe.

.

Signature: Printed Name Date

Unit: City State _ .

# Privacy Statement

The information obtained in the completion of this form will be used to provide the information required to complete the above-mentioned research goals.

Note: this form has been translated from the Arabic copy in order to make it clearer for non-Arabic speakers.
